# The Effects of Brown Algae-Derived Monosaccharide L-Fucose on Lipid Metabolism in C57BL/6J Obese Mice

**DOI:** 10.3390/nu12123798

**Published:** 2020-12-11

**Authors:** Xiao Yuan, Tomohiko Nakao, Hina Satone, Kazuyuki Ohara, Yuri Kominami, Miho Ito, Teruki Aizawa, Tomoya Ueno, Hideki Ushio

**Affiliations:** 1 Department of Aquatic Bioscience, Graduate School of Agricultural and Life Sciences, University of Tokyo, Tokyo 113-8657, Japan; yuanxiao_bruce@foxmail.com (X.Y.); nakao-tomohiko839@g.ecc.u-tokyo.ac.jp (T.N.); asatone@mail.ecc.u-tokyo.ac.jp (H.S.); ohr@showagakuin.ac.jp (K.O.); akomi@mail.ecc.u-tokyo.ac.jp (Y.K.); 2 Yaizu Suisankagaku Industry Co., Ltd., 5-8-13 Kogawa-shimmachi, Yaizu, Shizuoka 425-8570, Japan; mi-ito@yskf.co.jp (M.I.); t-aizawa@yskf.co.jp (T.A.); t-ueno@yskf.co.jp (T.U.)

**Keywords:** L-fucose, intra-abdominal fat, obesity, adipogenesis, metabolic diseases

## Abstract

Obesity is a global public health problem and a risk factor for several metabolic disorders as well as cancer. In this study, we investigated the effects of L-fucose on lipid metabolism through chronic and acute in vivo experiments in mice. In the chronic test, mice were fed a high-calorie diet (HCD) containing 0.0001%, 0.001%, 0.01%, and 0.1% L-fucose for one month. The L-fucose supplementation inhibited body weight and visceral fat mass gain in HCD-fed mice. The results of the acute test showed that L-fucose increased the ratio of serum high molecular weight adiponectin and enhanced glucose and lipid catabolism. Furthermore, L-fucose also decreased the expression of adipogenic genes (peroxisome proliferator-activated receptor γ and cluster of differentiation 36). In conclusion, this study provides a new approach to combat obesity and the related diseases.

## 1. Introduction

Obesity, characterized by excessive fat accumulation in the body, is a chronic disease and a global public health problem. This disease is closely related to genetic, endocrine, and psychological factors, as well as poor diets. Obesity is also widely regarded as a risk factor for various chronic metabolic diseases including type 2 diabetes, dyslipidemia, hyperglycemia, and/or hypertension [1]. Furthermore, obesity can also cause musculoskeletal disorders and increase the risk of cancers such as colorectal, breast, and endometrial cancer [2].

The adipose tissue secretes various adipokines to maintain metabolic homeostasis. Obesity is associated with dysregulated secretion of adipokines [3]. Adiponectin is an adipokine that enhances insulin sensitivity and stimulates fatty acid oxidation by promoting the AMP-activated protein kinase (AMPK) pathway [4]. The AMPK signaling pathway is considered a physiological energy control system [5]. Plasma adiponectin levels are decreased in the state of obesity and insulin resistance in high-fat diet-induced obese mice [6]. In the serum, there are three types of adiponectin oligomeric complexes, including trimer (low molecular weight, LMW, 67 kDa), hexamer (medium molecular weight, MMW, 140 kDa), and multimer (high molecular weight, HMW > 300 kDa) [7,8]. High molecular weight adiponectin is considered the most biologically active form [9]. A reduction in HMW adiponectin increases the risk of metabolic syndrome, a metabolic disorder associated with obesity, hyperlipidemia, diabetes, etc. [10,11]. Peroxisome proliferator-activated receptor γ (PPARγ) and cluster of differentiation 36 (CD36) are two important lipid metabolism-related genes that affect the cellular uptake of fatty acids and adipocyte differentiation [12,13].

Seaweeds are a crucial part of marine ecosystems and living resources. In recent years, numerous bioactive compounds have been isolated from different seaweed species, such as fatty acids, sterols, carotenoids, and polysaccharide dietary fibers. The presence of various bioactive compounds in seaweeds makes them highly applicable in the food supplement industries [14]. L-fucose is a hexasaccharide deoxysugar with the chemical formula C6H12O5, lacking oxygen at carbon 6 in the galactose. It is a structural monosaccharide from the brown algal polysaccharide, fucoidan. Fucoidan is an L-fucose-containing sulfated polysaccharide (FCSP), which can be extracted from the cell walls of brown algae species such as *Laminaria* and *Sargassum* [15]. Kim et al. reported that brown algae-derived fucoidan exhibits anti-obesity effects and inhibits fat accumulation in high-fat diet-induced obese mice [16]. Fucoidan also inhibits the differentiation of 3T3-L1 adipocytes [17]. It has been shown that L-fucose effectively improves obesity caused by a high-fat diet in mice [18] and cannot be metabolized as a source of energy [19]. The specific molecular mechanisms underlying the anti-obesity effects of L-fucose have not yet been fully elucidated. Since fucoidan is a heteropolysaccharide, the monosaccharide composition and sequences drastically affect its functional properties [20], rendering the quantitative control of its effects during application challenging. However, the fucoidan-derived monomer L-fucose is suitable as a functional food use because of its relatively high chemical stability and applicability in a purified form. Moreover, L-fucose is presumably safe for human oral administration, since human babies ingest monosaccharides via breast milk [21].

Male C57BL/6J mice have been reported to be more susceptible to visceral obesity compared to females [22]. Therefore, we selected male C57BL/6J as the animal model for research on obesity. The present study was designed to investigate the potential benefits of L-fucose on body weight in high-calorie diet-induced obese mice through an in vivo chronic test. Furthermore, the molecular mechanisms including obesity-related genes were also evaluated through an in vivo acute test to advance the understanding of the beneficial effects of L-fucose on obesity.

## 2. Materials and Methods

### 2.1. Animals and Diets

Six-week-old C57BL/6JJcl male mice (approximately 20 g in body weight, CLEA, Tokyo, Japan) were housed in a temperature and humidity-controlled laboratory animal room and entrained to a 12 h light-dark cycle with food and clean water available ad libitum.

For the chronic test, the American Institute of Nutrition-93 purified diets (AIN-93) were used as the basic diet. The high-calorie diet (HCD) contained 20% casein, 20% α-cornstarch, 9.625% β-cornstarch, 5% cellulose, 10% sucrose, 30% soybean oil, 1% AIN-93 vitamin mix, 3.5% AIN-93 mineral mix, 0.375% L-cystine, and 0.5% choline chloride (Oriental Yeast, Tokyo, Japan). The lipid ratio of this composition was 30%. The specific composition of the high-calorie diets containing L-fucose (Tokyo Chemical Industry, Tokyo, Japan) is shown in Table 1. The high-calorie diet was fed to induce obesity after adaptive feeding for one week. Obese mice were then randomly divided into six groups (*n* = 6) that were fed high-calorie diets containing 0.0001%, 0.001%, 0.01%, or 0.1% L-fucose, and control diet for 30 days. The body weight of the mice was measured daily.

For the acute test, after adaptive feeding for one week, the mice were randomly divided into three groups: blank control, glucose, and L-fucose groups (*n* = 5). Mice were fasted overnight prior to the experiment while ensuring access to drinking water. At the beginning of the trial, the blank control, glucose, and L-fucose groups were administered 0.5 mL of distilled water, 4 mg/mL glucose, or 4 mg/mL L-fucose solution per individual, respectively, through a stainless-steel feeding needle.

The animal experimental design and study were performed following the institutional protocol approved by the University of Tokyo (#P18-086H02).

### 2.2. X-ray Scan

In the chronic test, the visceral and liver fat amounts were measured once a week under continuous anesthesia with isoflurane (Wako Pure Chemical Industries, Tokyo, Japan) using a Latheta X-ray CT scanner (LCT-100, HITACHI, Tokyo, Japan). The X-ray CT images of the mice were obtained and analyzed using Latheta software v3.00 (HITACHI, Tokyo, Japan).

### 2.3. Blood and Tissue Collection

In the acute test, the blank control group was immediately sacrificed by cervical dislocation after oral administration with distilled water. The whole blood samples of mice belonging to the blank control group were collected from the heart. In the glucose and L-fucose groups, the animals were sacrificed by cervical dislocation after resuming feeding for 24 h, and whole blood samples from both groups were collected from the heart. All whole-blood samples were left to clot at room temperature for 4 h. The serum was separated from the clotted whole-blood samples by 3500 rpm centrifugation at 4 °C for 15 min and stored at −80 °C. The tissues (liver, skeletal muscle, and adipose) were collected after blood collection, soaked in RNA-later solution (Thermo Fisher Scientific Japan, Tokyo, Japan) at 4 °C for one day, and then used for total RNA extraction.

### 2.4. Biochemical Analysis of Serum

In the chronic test, mice were fasted overnight before blood collection. Whole blood was collected from the tail vein once a week. Fasting glucose levels were measured using a blood glucose meter (Glutest neo alpha, GT-1830; Arkray, Kyoto, Japan). Serum insulin was measured using a Mouse Insulin ELISA kit (FUJIFILM Wako Shibayagi, Gunma, Japan). The concentrations of serum total cholesterol (T-CHO) and non-esterified fatty acid (NEFA) were assayed using a 7180 Clinical Analyzer (HITACHI, Tokyo, Japan) in the 0.0001%, 0.001%, 0.01%, and 0.1% L-fucose-administered and control groups.

### 2.5. SDS-PAGE and Western Blotting

Serum samples were subjected to electrophoresis on a sodium dodecyl sulfate polyacrylamide gel (SDS-PAGE, Multi gel II mini, CosmoBio, Tokyo, Japan) under non-reducing or reducing conditions. Serum samples were diluted 30-fold with 1 × PBS and prepared in 4 × Laemmli sample buffer (Bio-Rad, Tokyo, Japan) without (non-reducing) or with (reducing) dithiothreitol (DTT, 50 mM). Proteins were transferred to a low-fluorescence PVDF membrane (TransBlot Turbo, Bio-Rad, Tokyo, Japan) using a Trans-Blot Turbo Blotting System (Bio-Rad, Tokyo, Japan). After the transfer, the membrane was blocked with Tris-buffered saline (pH 7.4) containing 5% BSA, 20 mM Tris, and 150 mM NaCl (5% BSA-TBS) at room temperature (25 °C) for 2 h. After washing three times, the membrane was incubated at 4 °C overnight with the primary antibody (Rabbit Anti-Adiponectin 1:1000 *v*/*v*, Ab3455, Abcam, Tokyo, Japan). After washing three times with TBS supplemented with 0.1% Tween-20 (TBST), the membrane was incubated with a secondary antibody (Goat Anti-Rabbit IgG (H + L), 1:10,000 *v*/*v*, Alexa Fluor 680, Abcam, Tokyo, Japan) at room temperature in the dark for 1 h. Finally, after three washes with TBST, the fluorescence intensity of adiponectin was detected using a LI-COR Odyssey (LI-COR, Lincoln, NE, USA) imaging system.

### 2.6. Total RNA Isolation

Total RNA was extracted from mouse tissue samples (liver tissue 50 mg; adipose tissue 100 mg; skeletal muscle 50 mg) using an RNeasy Plus Universal Mini Kit (Qiagen, Tokyo, Japan) following the manufacturer’s protocol. The purified total RNA was stored at −80 °C until further use.

### 2.7. Transcriptome Analysis

Total RNA concentration and RNA integrity (RNA integrity number equivalent; RINe) from liver tissue were measured using a Qubit 2.0 fluorometer (Thermo Fisher Scientific, Tokyo, Japan) and an Agilent TapeStation 2200 System (Agilent Technologies, Tokyo, Japan). High-quality RNA samples were used for RNA-Seq analyses. The RNA-Seq analyses were performed by Eurofins Genomics (Eurofins Genomics, Tokyo, Japan) with eight groups of RNA samples (glucose and L-fucose oral administration group, four samples per group, diluted to 5.5 μg/55 μL) using a HiSeq2500 system (Illumina, San Diego, CA, USA). The original sequence reads were cleaned and mapped to the mouse genome gene reference (GRCm38.mm10) using QIAGEN’s CLC Genomics Workbench v.11.0.1 (https://digitalinsights.qiagen.com/). Differentially expressed genes (DEGs) were identified between the glucose and L-fucose oral administration group using the reads per kilobase per million mapped reads (RPKM) values of corresponding transcripts. The filtering standard was set to more than a 1.5-fold change, and FDR *p*-value ≤ 0.05. The DEGs containing up- and downregulated genes were used for gene ontology (GO)-term analysis using the Database for Annotation, Visualization, and Integrated Discovery (DAVID, https://david.ncifcrf.gov/) tools [23,24] for their functional classification.

### 2.8. Quantitative Real-Time PCR

Total RNA was reverse-transcribed was using a GeneAmp PCR System 9700 (Thermo Fisher Scientific, Tokyo, Japan) with a PrimeScript RT reagent Kit (TAKARA, Tokyo, Japan). RNA concentration was measured by UV spectrophotometry (NanoPhotometer P300, Implen, Germany). Then 2 μg of total RNA from each sample was premixed with 1 μL gDNA Eraser Genomic DNA and 2 μL 5 × gDNA Eraser Buffer. The pre-mixture was added to 10 μL RNase-free water. Genomic DNA was eliminated at 42 °C for 2 min using a PCR system, premixing 1 μL PrimeScript RT Enzyme Mix I, 4 μL 5 × PrimeScript Buffer 2, and 4 μL RT Primer Mix. Then, 10 μL of the solution from the elimination reaction was added to the pre-mixture. Reverse transcription was performed at 37 °C for 15 min, followed by a cycle at 85 °C for 5 s. The cDNA samples were stored at −80 °C. TaqMan Gene Expression Assays (PPARγ: Mm00440940_m1, CD36: Mm00432403_m1, and β-actin: Mm00607939_s1, Thermo Fisher Scientific Japan, Tokyo, Japan) were diluted to 1/5, and cDNA samples were diluted to 1/20 with nuclease-free water. Two microliters of cDNA samples were mixed with 1 μL TaqMan Gene Expression Assay, 7 μL nuclease-free water, and 10 μL TaqMan Fast Advanced Master Mix. Subsequently, the mixtures were transferred to each well of a 96-well plate. The real-time PCR reaction was performed using the ABI PRISM 7300 system (Thermo Fisher Scientific Japan, Tokyo, Japan) with the following cycling conditions: 50 °C for 2 min, 95 °C for 20 s, followed by 40 cycles at 95 °C for 3 s, and 60 °C for 30 s.

### 2.9. Statistical Analysis

All data are expressed as mean ± standard error of the mean (SEM). Statistical analysis was performed using the GraphPad Prism 8 (v8.0.1) software. One-way analysis of variance (ANOVA) followed by Dunnett’s multiple comparisons was used to detect the significance of the data. *p*-values < 0.05 were considered statistically significant.

## 3. Results

### 3.1. L-Fucose Inhibits Body Weight and Liver Fat Mass Gains in High-Calorie Diet-Fed Mice

**Figure 1:**
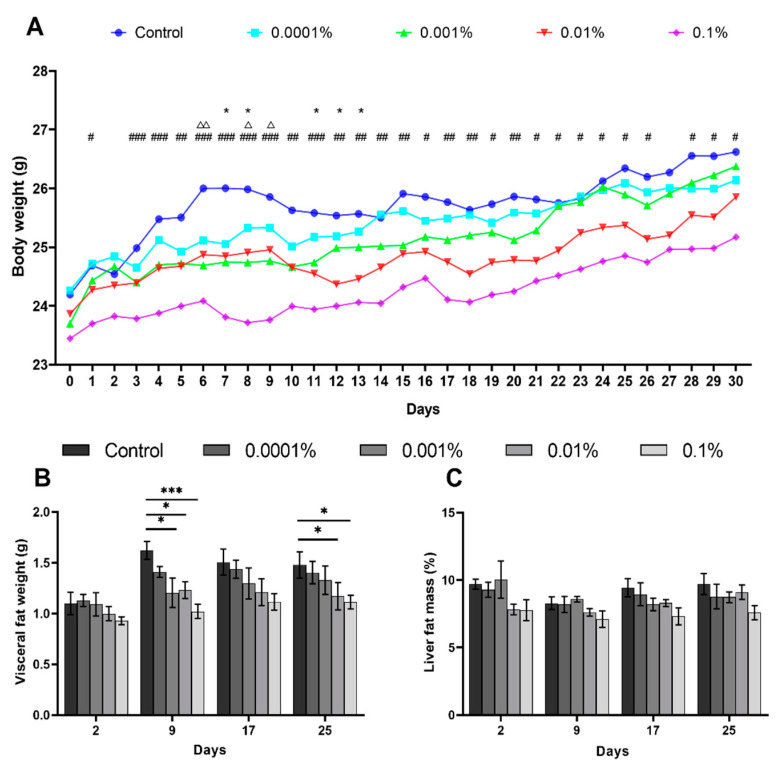
L-Fucose suppresses fat accumulation in high-calorie diet-induced obese mice. (**A**) Body weight gains in high-calorie diet-induced obese mice (*n* = 6) fed either a control high-calorie or high-calorie diet with 0.0001%, 0.001%, 0.001% and 0.1% L-fucose form day 0 to 30 (△ [Control vs. 0.001%], * [Control vs. 0.01%], # [Control vs. 0.1%]: *p* < 0.05; △△, ##: *p* < 0.01; ***, ###: *p* < 0.001). (**B**) Visceral fat weight and (**C**) liver fat mass of mice detected by CT-scan (Day 2, 9, 17, 25, *: *p* < 0.05, ***: *p* < 0.001).

In order to evaluate the effect of L-fucose on HCD-fed mice, we first performed body weight and X-ray scan tests in a one-month chronic experiment. In the chronic test, we found no significant difference in food intake between the control diet group and L-fucose-treated groups (data not shown). The body weight of mice increased gradually in all groups. The statistical analysis showed that the weight gain was significantly lower in the 0.001%, 0.01%, and 0.1% L-fucose groups than in the control group over the tested period, especially in the 0.1% L-fucose group (Figure 1A). The X-ray CT scan results showed that the visceral fat weight of the 0.1% and 0.01% L-fucose-administered groups was significantly lower than that of the control group on days 9 and 25 (Figure 1B). However, the liver fat mass of mice remained nearly unchanged (Figure 1C). There was no significant difference between the L-fucose treatment group and control in the fasting glucose and insulin levels (Supplementary Figure S1A,B). Although we observed that the total cholesterol level in the 0.1% L-fucose group was significantly lower than in the control group on day 21, we found no major change for other fucose treatment groups in the serum total cholesterol and NEFA levels (Supplementary Figure S2A,B).

### 3.2. L-Fucose Promotes Adiponectin Multimerization

**Figure 2:**
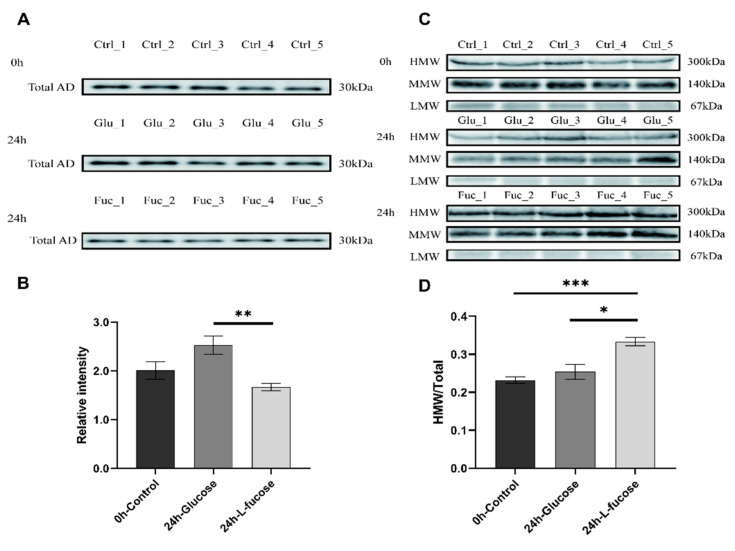
L-Fucose decreases the total adiponectin and changes the composition of multimeric serum adiponectin. (**A**) Western blot analysis of total adiponectin protein levels from serum of acute glucose and L-fucose-fed mice. (Total AD: total adiponectin, 30 kDa, *n* = 5). (**B**) Relative intensity of total serum adiponectin. (**C**) Western blot analysis of multimeric adiponectin protein levels from serum of acute glucose and L-fucose-administered mice (HMW: high molecular weight, ~300 kDa; MMW: medium molecular weight, ~140 kDa; LMW: low molecular weight, ~67 kDa, *n* = 5). (**D**) Ratio of serum HMW adiponectin to total adiponectin (*: *p* < 0.05, **: *p* < 0.01 ***: *p* < 0.001).

Serum adiponectin levels in mice were detected using Western blotting. Total adiponectin (30 kDa) levels were confirmed in the reducing condition (Figure 2A). The levels of total adiponectin were reduced by the L-fucose acute administration after 24 h (Figure 2B). In the non-reducing condition, we detected three different molecular weight bands: high molecular weight (HMW, ~300 kDa), medium molecular weight (MMW, ~140 kDa), and low molecular weight (LMW, ~67 kDa) of adiponectin (Figure 2C). Initially, the mean HMW adiponectin ratio was 23.2% (SEM 0.0088). After 24 h of acute oral administration of L-fucose, the values of HMW adiponectin in the L-fucose-administered group tended to increase. The mean serum HMW adiponectin ratios in the glucose and L-fucose groups were 25.4% (SEM 0.020) and 33.3% (SEM 0.011), respectively (Figure 2D). The serum HMW adiponectin ratio in the L-fucose group was significantly higher than in the glucose group (*p* < 0.01).

### 3.3. L-Fucose Enhances Glucose and Lipid Catabolism

**Figure 3:**
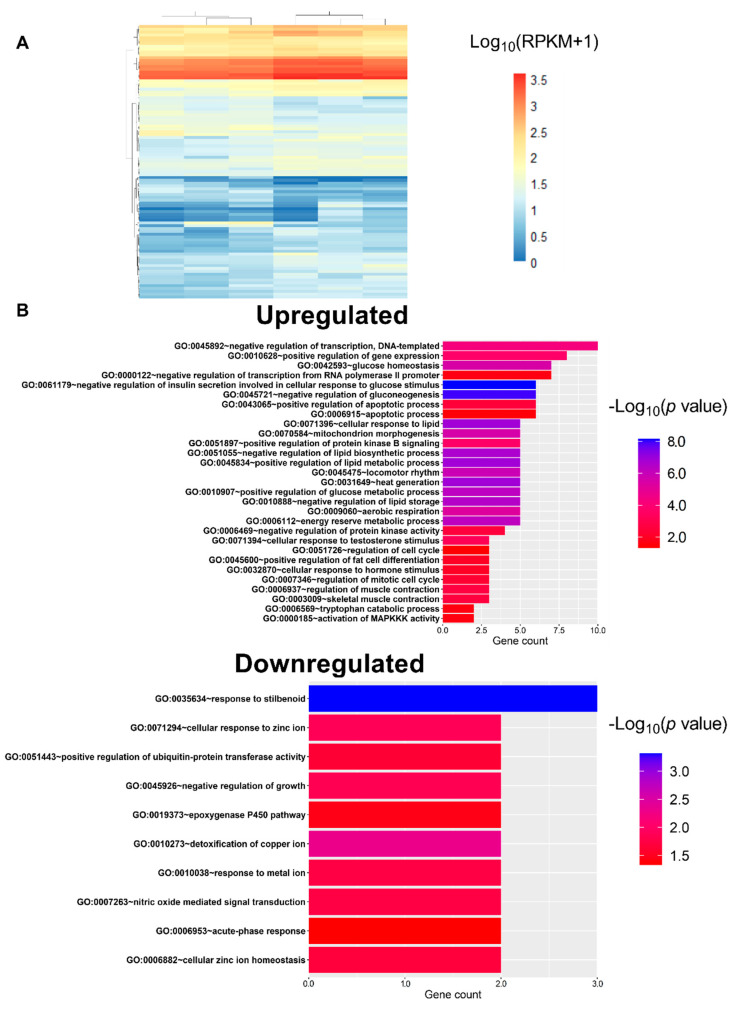
L-Fucose affects the energy metabolism of mice. (**A**) Heat map of expression values of DEGs between acute glucose and L-fucose-treated mice. Every sample correspond to a column, and every DEG to a row. DEGs were clustered by Log_10_(RPKM + 1) value, orange indicates highly expressed genes, blue indicates genes with lower expression. (**B**) Enrichment of GO biological processes terms using DEGs detected by DAVID. The ordinate shows the enriched GO term, and the abscissa shows the enriched differential gene count in the GO term. The -Log_10_(*p* value) was used to measure the significance.

High-throughput sequence data of eight samples from the liver (glucose and L-fucose treatment groups, four samples each) were generated using HiSeq2500. These reads were further analyzed using the CLC Genomics Workbench v.11.0.1, and the GRCm38 (mm10) reference gene set was chosen for mapping. The gene expression levels of each identified gene are represented as RPKM. At 24 h we identified DEGs in the liver tissue with a significant difference in the L-fucose group compared to the glucose group. We obtained a total of 96 DEGs from the analyses with fold change > 1.5, and *p*-value ≤ 0.05 (Figure 3A). The up- or downregulated genes were uploaded to the DAVID (https://david.ncifcrf.gov/) to analyze gene-annotation enrichment. The upregulated genes in L-fucose group compared to glucose group were enriched in sequences linked to biological processes related to insulin secretion, glucose and lipid catabolism, and lipid storage. The downregulated genes were instead enriched in sequences linked to biological processes related to the steroid hormone stimulus and cell growth (Figure 3B).

### 3.4. L-Fucose Decreases the Gene Expression of Lipid Metabolism-Related Genes

**Figure 4:**
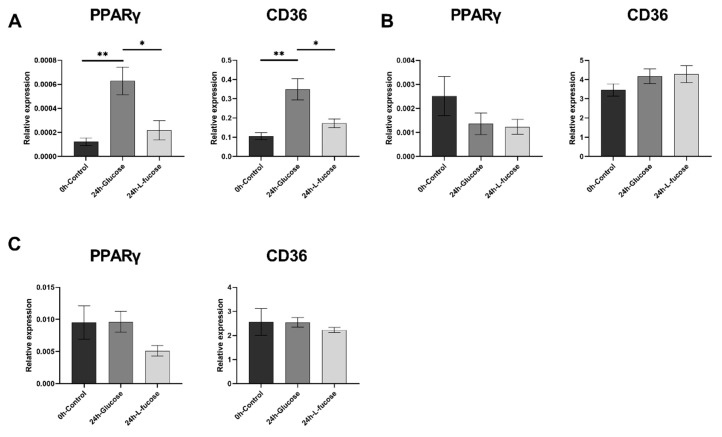
L-Fucose decreases the expression of adipogenic genes. Relative expression of PPARγ and CD36 in (**A**) liver, (**B**) skeletal muscle, and (**C**) adipose tissue of glucose and L-fucose-treated mice (*: *p* < 0.05, **: *p* < 0.01, The relative mRNA levels are expressed as the mean 2^−ΔΔCt^ relative to the control β-actin).

Having observed the effect of L-fucose on lipid metabolism by transcriptome analysis, we next analyzed the topic genes using quantitative real-time PCR to determine whether L-fucose could affect adipogenic gene expression. We determined the relative expression levels of lipid metabolism-related genes (PPARγ and CD36) from skeletal muscle, liver, and adipose tissues. After 24 h of oral glucose administration, the expression of PPARγ and CD36 genes in the liver increased. The expression of PPARγ and CD36 genes was significantly lower (*p* < 0.05) after L-fucose administration compared with glucose administration (Figure 4A). In the skeletal muscle, PPARγ gene expression levels showed a decreasing trend over time in both the glucose and L-fucose groups (Figure 4B), but no difference was observed in CD36 gene expression levels. Finally, in the adipose tissue, the gene expression of PPARγ in the L-fucose group tended to be lower than in the glucose group after 24 h of treatment, while we observed no effect on CD36 gene expression (Figure 4C).

## 4. Discussion

In the present study, we confirmed the anti-obesity effect of L-fucose using long-term chronic tests and determined the possible molecular mechanisms using acute tests in mice. Our results showed that L-fucose suppressed body weight and liver fat mass gain in HCD-induced obese mice, increasing the ratio of serum HMW adiponectin. Furthermore, it enhanced lipid catabolism and decreased lipid metabolism-related gene expression.

The HMW/total adiponectin ratio has been reported as a useful biomarker to predict metabolic syndrome [25,26]. Here, L-fucose promoted the multimerization of adiponectin, which reduces the risk of metabolic syndrome-related morbidities such as non-alcoholic fatty liver disease (NAFLD) [27]. While L-fucose might further activate the AMPK signaling pathway to enhance insulin sensitivity and stimulate fatty acid oxidation [28], here, L-fucose had no effect on serum glucose levels and insulin resistance, consistent to with the results from a previous study [18]. Our results also showed that L-fucose promoted the biological processes related to lipid and glucose consumption such as lipolysis and glycolysis, and inhibited lipogenesis. Thus, obesity can be counteracted by reducing energy production and increasing energy expenditure.

PPARγ, an adipogenic marker protein, is a ligand-activated nuclear hormone receptor, that exerts critical regulatory roles in adipogenesis [12]. PPARγ has been identified as a target for obesity treatment [29]. CD36 is a membrane glycoprotein belonging to the class B scavenger receptor family which is expressed in platelets, mononuclear phagocytes, adipocytes, hepatocytes, myocytes, and some epithelial cells [13]. As a fatty acid translocase, CD36 plays a crucial role in lipid transport and metabolism [30]. PPARγ regulates the gene expression of CD36 in the liver, muscle, and adipose tissue, and CD36 is generally used as a reporter gene of PPARγ [31,32,33]. Berberine, an isoquinoline alkaloid inhibits the expression of adiponectin through the inhibition of PPARγ transcriptional activity and promotes the multimerization of adiponectin [34], thus reducing adipose tissue mass [35]. The present results are consistent with those reported for berberine. Our findings suggest that L-fucose may prevent adipogenesis by inhibiting of PPARγ activity and stimulate fatty acid oxidation by promoting adiponectin multimerization. Furthermore, the reduction in PPARγ expression would result in a decrease in total serum adiponectin. The increased energy consumption, via heat generation and lipid catabolism, leads to increase fat consumption and reduce fat accumulation. The combination of decreased fat synthesis and increased fat consumption reduces the amount of fat accumulated. However, we found that L-fucose did not affect serum glucose and insulin levels. A possible explanation is that the effects on glucose may be L-fucose dose-dependent and therefore require further investigation with high-dose treatment.

L-Fucose is a monosaccharide obtained from the brown algae polysaccharide, fucoidan, which can be easily extracted from brown algae [36]. It is also considered safe for human oral administration [21]. Recently, L-fucose has been reported to ameliorate high-fat diet-induced obesity and influence the function of intestinal flora in C57BL/6 mice. However, the specific molecular mechanism has not yet been reported [18]. In this study, we demonstrated the effects of L-fucose on the transcriptional levels of lipid-related genes in C57BL/6 mice. L-Fucose might promote the multimerization of adiponectin, increase energy consumption, and reduce fat accumulation. Although the present results suggest that L-fucose could be used as a dietary supplement to prevent obesity, further studies are needed to validate this finding.

While the current study confirmed the transcriptional effects of L-fucose in mice, there are some limitations as well. First, in our study, the effects on body weight and liver fat content of L-fucose were observed after chronic administration, whereas the effects on the transcriptional effects of L-fucose were observed after an acute administration. Thus, we cannot exclude the possibility that other factors might be determinants for obesity prevention. Second, the mechanism underlying the increase in HMW adiponectin is still unclear. Previous studies have shown that adiponectin glycosylation affects the stability of HMW and MMW complexes [37,38,39,40]. L-Fucose might in fact affect adiponectin glycosylation. Further studies should be carried out to confirm the anti-obesity effect and safety of L-fucose. In conclusion, our results provide a basis for the application of brown algae-derived L-fucose for obesity-related disease prevention, but more detailed clinical validation studies are needed.

## Figures and Tables

**Table 1:** The composition of high-calorie experimental diets (g/kg diet).

	Control	0.0001%	0.001%	0.01%	0.1%
Casein	200	200	200	200	200
α-Cornstarch	200	200	200	200	200
β-Cornstarch	96.25	96.249	96.24	96.15	95.25
Sucrose	100	100	100	100	100
Cellulose	50	50	50	50	50
Soybean oil	300	300	300	300	300
AIN-93 mineral mix	35	35	35	35	35
AIN-93 vitamin mix	10	10	10	10	10
50% choline chloride	5	5	5	5	5
L-Cystine	3.75	3.75	3.75	3.75	3.75
L-Fucose	-	0.001	0.01	0.1	1

## Supplementary Materials

The following are available online at https://www.mdpi.com/2072-6643/12/12/3798/s1, Figure S1. Fasting serum glucose (A), and fasting serum insulin (B); Figure S2. Serum levels of total cholesterol (A) and non-esterified fatty acids (B).
