# Supplementary material for: The Effects of Brown Algae-Derived Monosaccharide L-Fucose on Lipid Metabolism in C57BL/6J Obese Mice
Source: Nutrients. 2020 Dec 11;12(12):3798. doi: 10.3390/nu12123798 (PMC7764515; doi:10.3390/nu12123798)
Supplement: Supplementary file 1 [file nutrients-12-03798-s001.pdf]

Supplementary Materials:

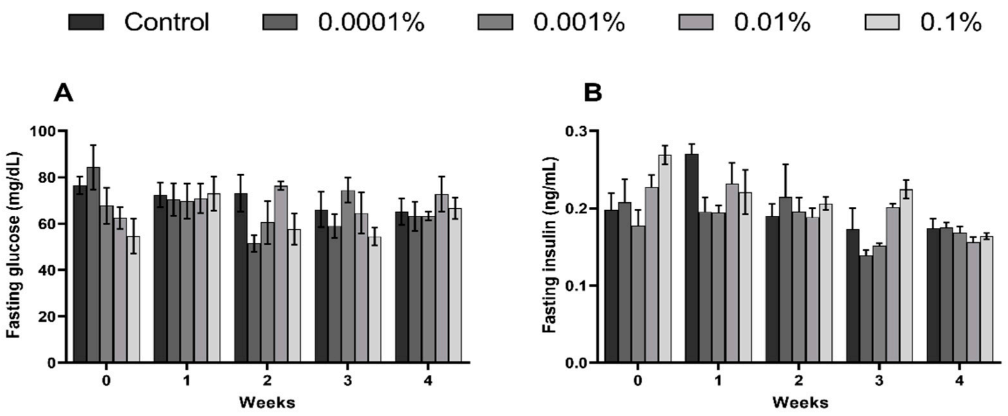

Figure S1. Fasting serum glucose (A), fasting serum insulin (B) were measured.

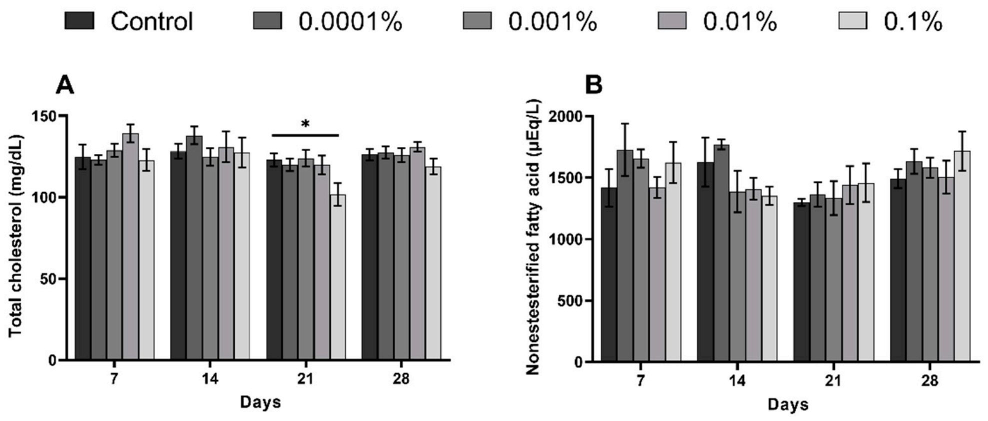

Figure S2. Serum level of total cholesterol (A) and non-esterified fatty acid fatty acid (B) were measured. (*: p<0.05).
